# *Mycoplasma hyorhinis *infection in gastric carcinoma and its effects on the malignant phenotypes of gastric cancer cells

**DOI:** 10.1186/1471-230X-10-132

**Published:** 2010-11-10

**Authors:** Hua Yang, Like Qu, Huachong Ma, Ling Chen, Wenbin Liu, Caiyun Liu, Lin Meng, Jian Wu, Chengchao Shou

**Affiliations:** 1 Key Laboratory of Carcinogenesis and Translational Research (Ministry of Education), Department of Biochemistry and Molecular Biology, Peking University Cancer Hospital & Institute, Beijing 100142, PR China; 2 Current address: Medical Testing College, Ningxia Medical University, Yinchuan 750004, PR China; 3 Department of General Surgery, Beijing Chaoyang Hospital, Beijing 100043, PR China; 4 Current address: Department of Health Science and Nursing, Wuhan Polytechnic University, Wuhan 430023, PR China

## Abstract

**Background:**

*Mycoplasma hyorhinis *infection has been postulated to play a role in the development of several types of cancer, but the direct evidence and mechanism remained to be determined.

**Methods:**

Immunohistochemistry assay and nested polymerase-chain reaction (PCR) were performed to examine the *mycoplasma hyorhinis *infection in gastric cancer tissues. Statistical analysis was used to check the association between mycoplasma infection and clinicopathologic parameters. Transwell chamber assay and metastasis assay were used to evaluate *mycoplasma hyorhinis*' effects on metastasis in vitro and in vivo. *Mycoplasma hyorhinis*-induced extracellular signal-regulated kinase (ERK) and epidermal growth factor receptor (EGFR) activation were investigated by Western blot.

**Results:**

My*coplasma hyorhinis *infection in gastric cancer tissues was revealed and statistical analysis indicated a significant association between mycoplasma infections and lymph node metastasis, Lauren's Classification, TNM stage, and age of the patients. *Mycoplasma hyorhinis *promoted tumor cell migration, invasion and metastasis *in vitro *and *in vivo*, which was possibly associated with the enhanced phosphorylation of EGFR and ERK1/2. The antibody against p37 protein of *Mycoplasma hyorhinis *could inhibit the migration of the infected cells.

**Conclusions:**

The infection of *m*y*coplasma hyorhinis *may contribute to the development of gastric cancer and *Mycoplasma hyorhinis*-induced malignant phenotypes were possibly mediated by p37.

## Background

Mycoplasma is one of the smallest living organisms isolated from nature, and can be cultured in a special medium. Three independent groups [1-3] had described *Mycoplasma hyorhinis *infection using PCR and enzyme-linked immunosorbent assay (ELISA) in gastric cancer, cervical condyloma tissues and ovarian cancer. *Mycoplasma sp*. infection was also documented in conventional renal cell carcinoma [4]. Our previous work demonstrated high infection rate of *mycoplasma hyorhinis *in gastric cancer tissues by immunohistochemistry (IHC) with PD4 monoclonal antibody against P37 protein of *Mycoplastma hyorhinis*. Mechanistically, mycoplasmal infection was found to inhibit apoptosis and induce malignant transformation of murine myeloid 32D cells [5]. We recently revealed that p37 promoted tumor cell motility, migration and invasion *in vitro *and enhanced tumor cell metastasis in C57BL/6 mice, which was mainly mediated by matrix metalloproteinase-2 (MMP-2) and the EGFR/MEK/ERK pathway [6]. Exogenous p37 protein was also found to alter gene expression profile, growth rate and morphology of prostate cancer cells [7]. All of these results suggest a possible link between mycoplasma infection and tumorigenesis, but the direct evidence remains elusive. In this study, the presence and the clinical significance of *mycoplasma hyorhinis *infection in gastric carcinoma tissues were analyzed with nested PCR and IHC assay. Meanwhile, the biological effects of *mycoplasma hyorhinis *infection on gastric cancer cells and the possible molecular mechanisms were investigated.

## Methods

### Cells and materials

The human MGC803 gastric carcinoma cells, derived from a poorly differentiated gastric cancer surgical specimen [8], were cultured in RPMI-1640 medium with 10% FCS. Human AGS gastric cancer cells and mouse B16F10 melanoma cells (both from American Type Culture Collection) were cultured in F-12 and RPMI-1640 medium plus 10% FCS, respectively. All culture media were from Invitrogen (Carlsbad, CA, USA). PD4, a mouse monoclonal antibody against p37 of *Mycoplasma hyorhinis*, was generated and characterized by our laboratory [9]. Pan-specific anti-mycoplasma antibody was a gift from the Beijing Institute of Basic Medical Sciences (Beijing, China). Transwell chambers (24-well) with 8.0-μm pore membranes were purchased from Corning (New York, NY, USA). Matrigel gel was purchased from Becton Dickinson (Franklin Lakes, NJ, USA). Antibodies to EGFR, pEGFR, ERK1/2 and pERK1/2 were purchased from Santa Cruz Biotechnology (Santa Cruz, CA, USA). Horseradish peroxidase (HRP)-coupled goat anti-mouse IgG and goat anti-rabbit IgG were from Zhongshan Biotechnology Company (Beijing, PR China).

### Gastric carcinoma specimens

Sixty-one freshly resected specimens from patients with gastric carcinoma were collected in the Beijing Cancer Hospital (Beijing, China). Informed consent was obtained from each patient and the study was approved and supervised by the Medical Ethics Committee of Peking University Cancer Hospital & Institute. Within 30 minutes of removal, cancer tissues (~0.5 cm^3^) were transported to laboratory in an icebox and processed on a petri dish in the fume hood by washing with 15 ml ice-cold phosphate-buffered saline (PBS) for three times, followed by slicing into 1 mm^3 ^cubes with a sterilized scissor. The sliced samples were then kept in cryo tubes and frozen at -80°C for later isolation of DNA.

### Preparation of conditional medium and *Mycoplasma hyorhinis *infection

Cell culture supernatant of MGC803 cells-infected by *Mycoplasma hyorhinis *was centrifuged at 3000 rpm for 5 min to remove cell debris, followed by centrifugation at 12,000 rpm for 1 hour. The pellet containing *Mycoplasma hyorhinis *was diluted with fresh medium and added to AGS cells culture for infection. After two weeks, efficiency of infection was verified by Western blot with PD4 antibody.

### Detection of *Mycoplasma hyorhinis *DNA with nested PCR

Total DNA was isolated from 10 mg of previously frozen gastric cancer tissue by digestion overnight in 0.5% Tween-20, 50 mM Tris (pH 8.5), 1 mM EDTA, and 200 mg/L proteinase K, followed by phenol/chloroform/isoamyl alcohol extraction and sodium acetate precipitation. DNA precipitates were washed with 70% ethanol, dried, and dissolved in 20 μL sterile H_2_O. A nested PCR kit (Clone-Gen; Wuxi Institute for Clone Genetic Technology, Jiangsu Province, PR China) was used according to the manufacturer's instructions to detect different mycoplasmas, including *Mycoplasma hyorhinis *and *Mycoplasma fermentans*. The nested PCR was performed with 1 to 2 μL of extracted DNA (~0.5 μg) from the tissues. The DNA was added to 15 μL of the reaction solution, including a pair of universal mycoplasmal primers (P1: 5'-ACACCATGGGAGCTGGTAAT-3', P2: 5-CTTCATCGACTTTCAGACCCAAGGCAT-3), dNTPs, Taq DNA polymerase, and PCR buffer, to a total volume of 25 μL. The DNA was denatured at 94°C for 2 min first, and then 93°C for 30 s, 55°C for 30 s, and 72°C for 60 s for 35 cycles and finally at 72°C for 10 min. The first PCR product was diluted 10 folds, and 5 μL of the diluted product was used as a template. A second sets of specific mycoplasmal primers annealing to gene sequences coding for evolutionarily conserved 16S rRNA of different mycoplasma species (*Mycoplasma hyorhinis*: P1:5'-CAAGATAAAATCATTTCCT-3', P2:5-AGTAATAGAAAGGAGCTTC-3; *Mycoplasma fermentans*: P1:5'-GAAGCCTTTCTTCGCTGGAG-3', P2: 5'-ACAAAATCATTTCCTATTCTGTC-3') were used with the same amplification conditions as described above. An internal control was introduced to exclude any false-negative test resulting from artificial causes. The PCR products were analyzed by agarose gel electrophoresis and DNA sequencing.

### Immunohistochemistry staining

Paraffin-embedded gastric tumor tissues were obtained from Department of Pathology of Peking University Cancer Hospital & Institute (Beijing, PR China). The tissue sections were stained with a pan-specific anti-mycoplasma antibody by IHC techniques according to the standard protocol [10] and the results were judged by two pathologists independently. (-) means no staining was observed in cells, (+) means less than 50% cells were observed to be stained, and (++) indicates that over 50% cells were stained. Normal mouse IgG was used as a negative control.

### Cell colony-forming assay

500-1000 indicated cells were cultured per well in six well plates. After 5 to 10 days, the cell colonies were fixed in acetone-methanol (1:1) and stained with 5% crystal violet, and the colonies (more than 50 cells of each aggregates or colonies which were visible by naked eyes) were counted.

### Cell migration and invasion assay

Cell migration assay was performed by using culture medium-treated 6.5 mm Transwell chamber with 8.0 μm polycarbonate membranes. According to the previous method [11], the bottom chamber was filled with 800 μL medium containing 10% FCS. Cells were harvested from tissue culture plates by serum-free medium, and then were seeded onto the top chamber of each Transwell at a density of 2×10^5^-1×10^6 ^cells/mL/well (100 μL/chamber) in serum-free medium. After incubation in a humidified incubator with 5% CO_2 _at 37°C for 24 hours, non-migratory cells were scraped off from the top of the Transwell with a cotton swab. The cells attached to the bottom side of the membrane were fixed by methanol, stained with 5% crystal violet, and counted under a light microscope.

The invasion assay was similar to the migration assay described above, except that the upper side of the membranes was coated with a uniform thickness (2 mm) of 100 μg Matrigel for 60 min at room temperature (RT) before the cells were added.

### Cell migration inhibition assay

The assay of migration inhibition was similar to the migration procedure described above, except that MGC803 cells were seeded onto the top chamber of each Transwell and different concentrations of normal rabbit IgG, rabbit anti-GST or rabbit anti-p37 antibody were added simultaneously. The concentrations of antibody were 20 μg, 50 μg, 100 μg or 200 μg per mL, respectively.

### Metastasis assay *in vivo*

The animal experiments were approved and supervised by the Medical Ethics Committee of Peking University Cancer Hospital & Institute. Female C57BL/6 mice (Vital River Laboratories, Beijing, PR China) were maintained under a germ-free conditions in the animal facility and used at 8-10 weeks of age, weighed 18-20 g. Uninfected or *Mycoplasma hyorhinis*-infected B16F10 cells were injected into C57BL/6 mice via tail lateral vein (2 × 10^5 ^cells per recipient) respectively. After 25 days, mice were sacrificed. The weight of lung was weighed and the number of metastatic tumor foci on the surface of mice's lung was counted. Meanwhile, the metastatic foci on livers and kidneys were also checked.

### Western blot analysis

To assess the effect of *Mycoplasma hyorhinis *on EGFR and ERK activation, cells were washed with PBS and harvested by centrifugation. Cells lysates were prepared as described previously [12]. Equal aliquot of the total protein were separated by SDS-PAGE and transferred onto nitrocellulose membrane. The membranes were probed with antibodies to pEGFR or pERK1/2 and HRP-conjugated IgG as secondary antibody. The enhanced chemiluminesence (ECL) system (Amersham-Pharmacia, NJ, USA) was employed in this experiment. The same membrane was then stripped and reprobed with anti-EGFR or ERK1/2 antibody to determine the total protein abundance.

### Statistical analysis

The data were analyzed using the χ2 test by SPSS 13.0 software (SPSS) and *P *< 0.05 was considered significantly.

## Results

### Mycoplasma infection in gastric carcinoma tissues

**Figure 1:**
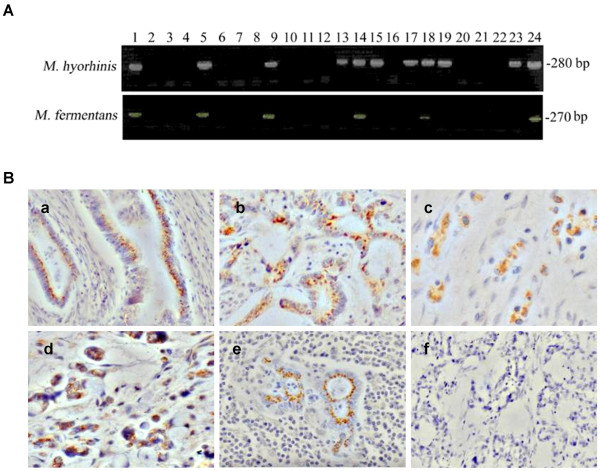
**Mycoplasma infection in gastric cancer tissues. A. **Nested PCR of mycoplasmal DNA amplified from human gastric cancer tissues. Universal primers were used for the first round PCR and *Mycoplasma hyorhinis*-specific primers or *Mycoplasma fermentans*-specific primers for second round PCR to amplify the mycoplasmal DNA. Lanes 1 and 2 are positive and negative controls, respectively. Lanes 5, 9, 14, 18, and 24 show results from tissues from which both *Mycoplasma hyorhinis *and *Mycoplasma fermentans *DNA were amplified. Lanes 13, 15, 17, 19, and 23 show results from tissues from which only *Mycoplasma hyorhinis *DNA was amplified. **B. **IHC staining of gastric cancer tissues with a pan-specific anti-mycoplasma antibody (400 ×). (a, b, c) well-differentiated, moderately differentiated and poorly differentiated adenocarcinomas, respectively. (d) mucous adenocarcinoma. (e) lymph node metastasis of moderately differentiated adenocarcinoma. (f) control staining with IgG.

To detect *Mycoplasma hyorhinis *infection in gastric cancer tissues, a nested PCR assay was performed. Thirty-nine samples (39/61, 63.9%) were positive for *Mycoplasma hyorhinis*; among them, nineteen (19/61, 31.1%) were also positive for *Mycoplasma fermentans *(Figure 1A). This finding was confirmed by sequencing of the PCR-amplified DNA sequences (data not shown). Statistical analysis of the relationship between PCR results and the data of clinical pathology and follow-up revealed that there was a significant association between mycoplasma infections, lymph node metastasis, Lauren's Classification, TNM stage and age of the patients (*P *< 0.05) (Table 1). There was more lymph node metastasis in mycoplasma infection group than in non-infected group. More mycoplasma infection was observed in diffuse-type gastric carcinoma than in the intestinal-type. The infection rate of mycoplasma in TNM stage III/IV was higher than in stage I/II. In addition, older patients were prone to be infected by mycoplasma.

**Table 1:** Detection of Mycoplasma DNA from Gastric Carcinoma Tissues by Nest- PCR and its correlation with clinicopathologic characteristics

	Differences		Correlation	
	
	χ2 value	*P *value	*R *value	*P *value
Lauren's Classification	5.584	0.018	0.303	0.018
lymph node metastasis	3.832	0.05	0.251	0.051
TNM stage	5.294	0.021	0.295	0.021
age	15.398	0.000	0.447	0.000

To further confirm the presence of mycoplasmas in gastric cancer tissues, we performed IHC staining on paraffin-embedded specimens from the same 61 patients using a pan-specific antibody against mycoplasma. Twenty-eight primary tumors (28/61, 45.9%) were scored as positive for mycoplasmas, 25 of which had also been found to be positive according to the results of nested PCR. Stained as brown granules, the mycoplasmas were localized mainly in the cytoplasm of cancer cells, as shown in Figure 1B.

### *Mycoplasma hyorhinis *promotes tumor cell colony formation, migration, and invasion in *vitro*

**Figure 2:**
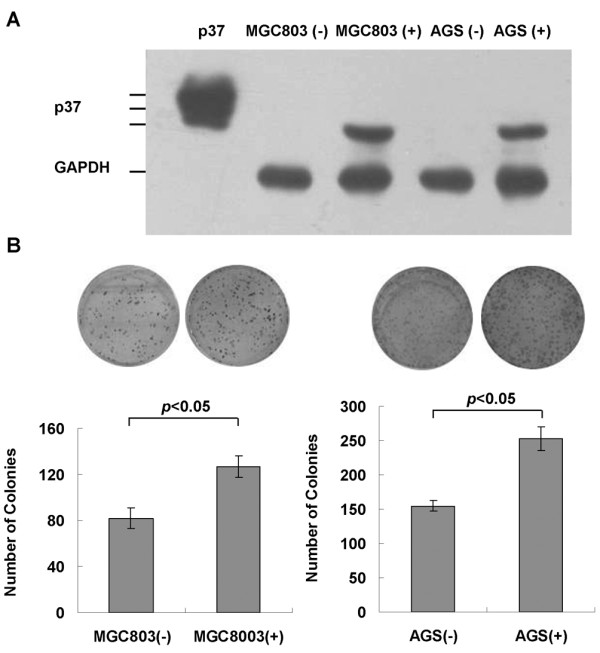
***Mycoplasma hyorhinis *promotes colony formation of cancer cells. A. **Verification of *Mycoplasma hyorhinis *infection. Mycoplasma-infected (+) and non-infected (-) MGC803 and AGS cells were harvested and cell lysates were subjected to Western blot analysis with PD4 antibody against p37 protein. Purified p37 protein [6] was used as positive control. **B. **Colony formation assay. 500-1000/well cells were cultured in 6 well plates for 5-10 days. The cell colonies were fixed by acetone-methanol (1:1) and stained with Crystal Violet. The numbers of colonies were counted and the graphs were the composite results from three independent experiments. There were more colonies in infected cells than non-infected cells (*P *< 0.05).

**Figure 3:**
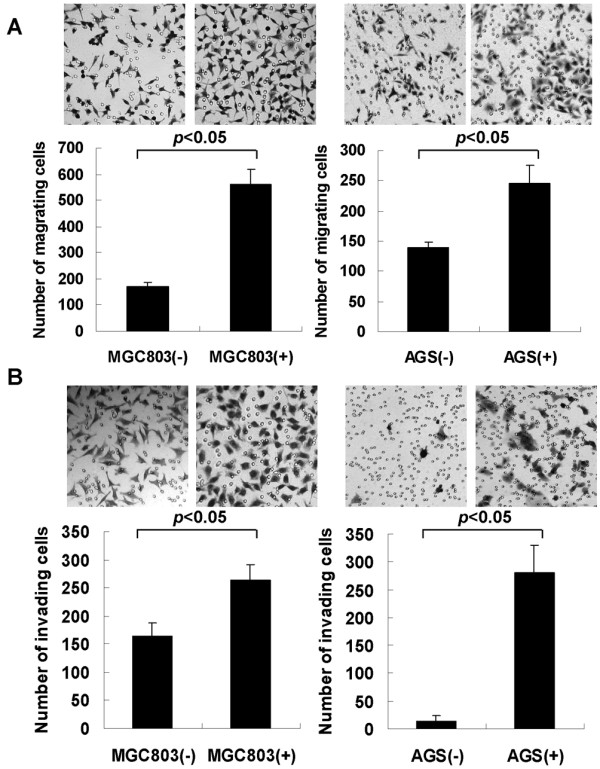
***Mycoplasma hyorhinis *promotes migration and invasion. **2 × 10^4^-1 × 10^5 ^cells were cultured in the top chamber of Transwell for 24-36 hours. After removing the cells on the top with a cotton swab, the cells attached on the bottom side of the membrane were fixed by methanol, stained with 5% crystal violet. The number of cells was counted. The graphs were the composite results from three independent experiments. **A. **Migration of uninfected cells (-) or infected cells (+). **B. **Invasion of uninfected cells (-) or infected cells (+). The number of migration cells and invasion cells of infected group were higher than those of uninfected group (*P *< 0.05).

After confirming *Mycoplasma hyorhinis *infection in gastric cancer tissues, we used the *Mycoplasma hyorhinis *to infect gastric cancer cell lines MGC803 and AGS to investigate the effects of the infection on these cells. After confirming the expression of p37 protein in infected MGC803 and AGS cells by Western blot (Figure 2A), colony formation assay was performed. More colonies were formed in infected cells than in non-infected cells (*P *< 0.05) (Figure 2B). Our previous study showed that p37 enhanced cancer cell migration and invasion *in vitro *[6]. Because p37 is a membrane lipoprotein of *Mycoplasma hyorhinis*, we predicted that, as the intact pathogen, *Mycoplasma hyorhinis *may have similar effects on such phenotypes. To test this, a modified Transwell chamber assay was employed and the result showed that cells' capacities of migration and invasion were significantly increased upon infection (*P *< 0.05) (Figure 3A,B).

### *Mycoplasma hyorhinis *stimulates cancer cell metastasis in C57BL/6 mice

**Figure 4:**
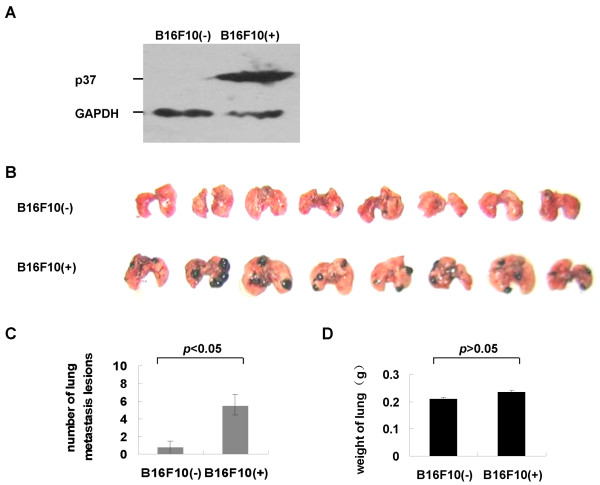
***Mycoplasma hyorhinis *stimulates tumor metastasis in C57BL/6 mice. A. **Verification of *Mycoplasma hyorhinis *infection in B16F10 cells by Western blot with PD4 antibody. **B. **Macroscopic documentation of lung metastasis lesions of mice injected with infected (+) or uninfected (-) B16F10 cells. **C. **The average numbers of metastatic lesions for each mouse. The infected group had more metastasis than the uninfected group (*P *< 0.05). **D. **The average weight of lungs of mice injected with B16F10 cells. No difference was observed (*P *> 0.05)

Next, in vivo metastasis assay was performed to better substantiate results from in vitro studies. However, AGS cells were poor in forming tumors in mice and MGC803 cells' metastasis potential was low (data not shown). In the previous study we had used p37 adenovirus-infected B16F10 melanoma to examine the metastatic lesions in the lung of C57BL/6 mice [6]. Herein similar approach was utilized. B16F10 cells were infected by *Mycoplasma hyorhinis*, which was verified by the expression of p37 protein (Figure 4A). The infected and uninfected B16F10 cells were injected into C57BL/6 mice via tail lateral vein, respectively. After twenty-five days, the number of metastatic lesions in the lung and liver of mice were counted. The metastatic foci in the lung increased significantly (Figure 4B,C; *P *< 0.05), but no difference in the weight of lungs was observed (Figure 4D) and no metastatic foci were formed in the livers or kidneys (data not shown). We then concluded that *Mycoplasma hyorhinis *infection stimulates tumor metastasis in C57BL/6 mice.

### *Mycoplasma hyorhinis *promotes phosphorylation of EGFR and ERK1/2

**Figure 5:**
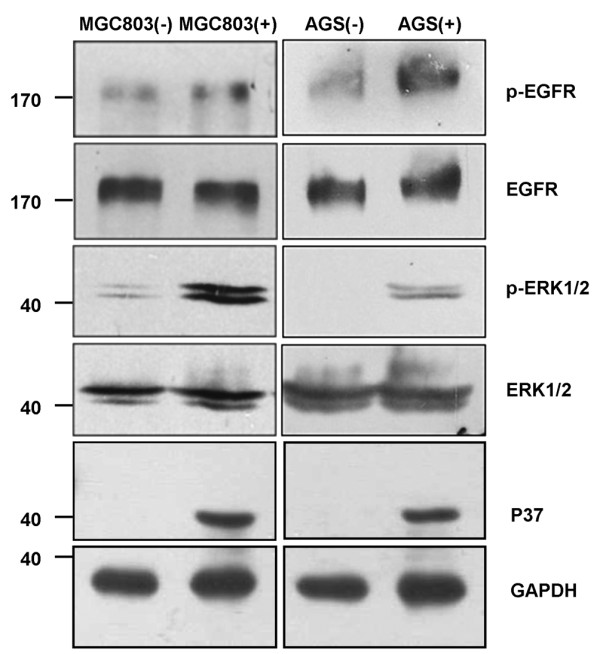
***Mycoplasma hyorhinis *stimulates EGFR and ERK1/2 phosphorylation in gastric cancer cells. **Lysates from *Mycoplasma hyorhinis *infected MGC803 (left panel) and AGS cells (right panel) were subjected to Western blot analysis with antibodies against pEGFR, EGFR, pERK1/2 and ERK1/2.

The p37-promoted the phosphorylation of EGFR and ERK1/2 has been noticed in our previous work [6], which may contribute to *Mycoplasma hyorhinis *infection-related phenotype. We examined the expression of pEGFR and pERK in *Mycoplasma hyorhinis*-infected MGC803 and AGS cells. The result showed that phosphorylation of both EGFR and ERK1/2 was substantially enhanced. Therefore *Mycoplasma hyorhinis *infection could activate EGFR and ERK1/2 (Figure 5).

### Antibody against p37 abrogates *Mycoplasma hyorhinis*-induced cell migration

**Figure 6:**
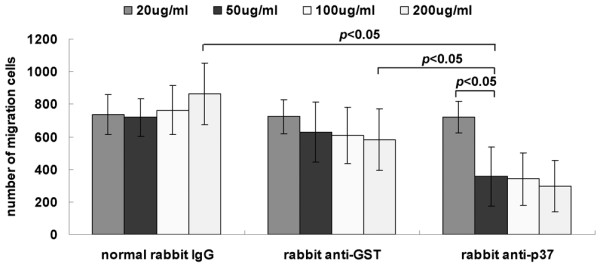
**Antibody against p37 blocks *Mycoplasma hyorhinis*-promoted cell migration. **Infected MGC803 cells were treated with indicated concentration of normal rabbit IgG, rabbit anti-GST or rabbit anti-p37 antibody, followed by Transwell chamber migration assay. Antibody against p37 inhibited *Mycoplasma hyorhini*-promoted migration with a dose-dependent manner (*P *< 0.05).

Although previous study suggested that p37 may facilitate *Mycoplasma hyorhinis*-induced tumor cell invasion, whether the invasion of tumor cell was directly mediated by p37 is unknown. We then performed a migration inhibition assay. As shown in Figure 6, the migration ability of *Mycoplasma hyorhinis-*infected cells was reduced dramatically after being treated with the antibody against p37 (≧50 μg/mL), but the normal rabbit antibody and GST antibody had no effect, even at high concentration (up to 200 μg/mL, *P *< 0.05), indicating *Mycoplasma hyorhinis*-induced cell migration was directly mediated by p37 protein.

## Discussion

Our previous work showed that there was high incidence of mycoplasma infection in gastric cancer tissues by IHC, which suggests a possible association between mycoplasma infection and tumorigenesis [13]. In the present study, we demonstrated the presence of mycoplasma in gastric cancer tissues through two independent methods: nested PCR assay and IHC staining. Positive correlation among different experimental methods confirmed that *Mycoplasma hyorhinis *and *Mycoplasma fermentans *were indeed positive in gastric carcinoma. Interestingly, we found that *Mycoplasma fermentans *infection in gastric cancer tissues was always accompanied by *Mycoplasma hyorhinis *infection, but *Mycoplasma hyorhinis *infection could exist independent of *Mycoplasma fermentans*. Therefore the result provides the first direct evidence of the presence of *Mycoplasma hyorhinis *in human gastric tumor tissues. We also analyzed the results of PCR detection (61 cases) with clinicopathologic data and noticed that mycoplasma infection had a close relationship with the lymph node metastasis, Lauren's Classification, TNM stage and age (*P *< 0.05). Compared to IHC, PCR possesses higher sensitivity, so the result of PCR may be more reliable to investigate the relationship between mycoplasma infection and gastric cancer's clinical manifestation and prognosis.

Long-term mycoplasma infection of cultured cells was associated with chromosomal instability and promoted malignant transformation [5,14,15]. Persistent infections with *mycoplasma genitalium *and *mycoplasma hyorhinis *induced malignant transformation of benign human prostate cells BPH-1 and increase migration and invasion [16]. Moreover, mycoplasma infection reduced p53 activation, activated NF-κB and cooperated with oncogenic Ras in the transformation of rodent fibroblast [17]. p37 is part of a homologous, high-affinity transport system on the membrane of *Mycoplasma hyorhinis *[18,19]. Other studies showed that p37 enhances tumor cell invasion *in vitro *[20-22], inhibits mammalian cell adhesion [12], and induces secretion of TNF-α from human peripheral blood mononuclear cells [23-25]. Recently, we demonstrated that p37 may promote gastric cancer cell invasion and metastasis by increasing the activity of MMP-2 and by inducing EGFR phosphorylation, therefore contributing to tumor metastasis upon *Mycoplasma hyorhinis *infection [6]. Based on these findings, herein we analyzed the phenotypes of MGC803 and AGS cells infected with *Mycoplasma hyorhinis*, and the results indicated that *mycoplasma hyorhinis *could also promote tumor cell migration and invasion *in vitro *and *in vivo*.

Blocking antibodies had been utilized to study the phenotypes of mycoplasma-infected cells. Dudler et al [18] revealed that MAb DD9 treatment could alter the mycoplasma metabolism and indirectly modify their interaction with the host cells. Moreover, MAb 243-5 was reported to reduce the lung metastasis of colon cancer in the nude mice model [19]. This antibody could specifically recognize a mycoplasmal protein with an MW of 47 kDa, which was obtained by immunization with *Mycoplasma arginini*-infected RPMI 4788 cells. PD4, a specific monoclonal antibody against p37, inhibited tumor-growth of MGC-803 cells in nude mice by inducing apoptosis and the proliferation of Ras-transformed cells [8,26,27]. Although *Mycoplasma hyorhinis *and p37 had long been predicted to be associated with increased invasiveness and tumor metastasis [6,19,21,22,28-30], the direct evidence, of which *Mycoplasma hyorhinis *promoting tumor cell invasion through the p37 protein, was missing. Our present work showed that *Mycoplasma hyorhinis *could promote tumor cell colony formation, migration and invasion. Furthermore, antibody against p37 inhibited the migration of *Mycoplasma hyorhinis*-infected tumor cells. These results suggested p37 is a critical molecule mediating *Mycoplasma hyorhinis *infection and promoting cell migration.

## Conclusions

In summary, we have successfully detected *Mycoplasma hyorhinis *and *Mycoplasma fermentans *in gastric cancer tissues, and for the first time, found the correlation between mycoplasma infection and clinicopathologic characteristics for gastric cancer patients. p37 is critical for promoting cancer cells' malignant phenotypes. Our present works reveal the potential roles of mycoplasma infection in gastric cancer development and highlight the close relationships among *Mycoplasma hyorhinis *infection, p37 and tumorigenesis.

## Competing interests

The authors declare that they have no competing interests.

## Authors' contributions

CS conceived and supervised the project; CS, LQ and HY designed the experiments; HY, HM, LC, WL, CL, LM and JW performed the experiments and collected data; CS, LQ and HY analyzed data; HY, LQ and CS wrote the manuscript. All authors read and approved the final manuscript.

## Pre-publication history

The pre-publication history for this paper can be accessed here:

http://www.biomedcentral.com/1471-230X/10/132/prepub

## References

[B1] Sasaki H Igaki H Ishizuka T Kogoma Y Sugimura T Terada M Presence of streptococus DNA sequence in surgical specimens of gastric cancer Jpn J Cancer Res 1995 86 791 794 7591953 10.1111/j.1349-7006.1995.tb03086.x PMC5920939

[B2] Kidder M Chan PJ Seraj IM Patton WC King A Assessment of archived paraffin-embedded cervical condyloma tissues for mycoplasma-conserved DNA using sensitive PCR-ELISA Gynecol Oncol 1998 71 254 257 10.1006/gyno.1998.5177 9826468

[B3] Chan PJ Seraj IM Kalugdan TH King A Prevalence of mycoplasma conserved DNA in malignant ovarian cancer detected using sensitive PCR-ELISA Gynecol Oncol 1996 63 258 260 10.1006/gyno.1996.0316 8910637

[B4] Pehlivan M Pehlivan S Onay H Koyuncuoglu M Kirkali Z Can mycoplasma-mediated oncogenesis be responsible for formation of conventional renal cell carcinoma? Urology 2005 65 411 414 10.1016/j.urology.2004.10.015 15708077

[B5] Feng SH Tsai S Rodriguez J Lo SC Mycoplasmal infections prevent apoptosis and induce malignant transformation of interleukin-3-dependent 32D hematopoietic cells Mol Cell Biol 1999 19 7995 8002 10567525 10.1128/mcb.19.12.7995 PMC84884

[B6] Gong MM Meng L Jiang BH Zhang JZ Yang H Wu J Shou C p37 from *Mycoplasma hyorhinis *promotes cancer cell invasiveness and metastasis through activation of MMP-2 and followed by phosphorylation of EGFR Mol Cancer Ther 2008 7 530 537 10.1158/1535-7163.MCT-07-2191 18347140

[B7] Goodison S Nakamura K Iczkowski KA Anai S Boehlein SK Rosser CJ Exogenous mycoplasmal p37 protein alters gene expression, growth and morphology of prostate cancer cells Cytogenet Genome Res 2007 118 204 213 10.1159/000108302 18000372

[B8] Xiao H Shou CC Dong ZW Induction of apoptosis in human gastric carcinoma cell line MGC-803 by monoclonal antibody PD4 Chin J Oncol 1998 20 31 33 10921052

[B9] Dong ZW Wan WH Li ZP Qiu WR Wei SM A monoclonal antibodies PD4 against gastric cancer cell line MGC803 Shengwu Huaxue Zazhi 1985 1 52 58

[B10] Meenakshi A Kumar RS Ganesh V Sivakumar N Immunohistochemical assay (IHC) to study C-erbB-2 status of breast cancer using monoclonal antibody CIBCgp185 Hum Antibodies 2003 12 123 127 15156100

[B11] Du CW Wen BG Li DR Peng X Hong CQ Chen JY Lin ZZ Hong X Lin YC Xie LX Wu MY Zhang H Arsenic trioxide reduces the invasive and metastatic properties of nasopharyngeal carcinoma cells *in vitro* Braz J Med Biol Res 2006 39 677 685 10.1590/S0100-879X2006000500015 16648906

[B12] Liu WB Zhang JZ Jiang BH Ren TT Gong MM Meng L Shou C Lipoprotein p37 from *Mycoplasma hyorhinis *inhibiting mammalian cell adhesion J Biomed Sci 2006 13 323 331 10.1007/s11373-005-9045-7 16328779

[B13] Huang S Li JY Wu J Meng L Shou CC Mycoplasma infections in different carcinomas World J Gastroenterol 2001 7 266 269 11819772 10.3748/wjg.v7.i2.266 PMC4723534

[B14] Tsai S Wear DJ Shih JW Lo SC Mycoplasmas and oncogenesis: persistent infection and multistage malignant transformation Proc Natl Acad Sci USA 1995 92 10197 10201 10.1073/pnas.92.22.10197 7479753 PMC40763

[B15] Zhang S Tsai S Lo SC Alteration of gene expression profiles during mycoplasma-induced malignant cell transformation BMC Cancer 2006 6 116 10.1186/1471-2407-6-116 16674811 PMC1559712

[B16] Namiki K Goodison S Porvasnik S Allan RW Iczkowski KA Urbanek C Reyes L Sakamoto N Rosser CJ Persistent exposure to Mycoplasma induces malignant transformation of human prostate cells PLoS One 2009 4 e6872 10.1371/journal.pone.0006872 19721714 PMC2730529

[B17] Logunov DY Scheblyakov DV Zubkova OV Shmarov MM Rakovskaya IV Gurova KV Tararova ND Burdelya LG Naroditsky BS Ginzburg AL Gudkov AV Mycoplasma infection suppresses p53, activates NF-kappaB and cooperates with oncogenic Ras in rodent fibroblast transformation Oncogene 2008 27 4521 4531 10.1038/onc.2008.103 18408766 PMC4526267

[B18] Dudler R Schmidhauser C Parish RW Wettenhall RE Schmidt T A mycoplasma high-affinity transport system and the *in vitro *invasiveness of mouse sarcoma cells EMBO J 1988 7 3963 3970 3208756 10.1002/j.1460-2075.1988.tb03283.x PMC454995

[B19] Ushio S Iwaki K Taniai M Ohta T Fukuda S Sugimura K Kurimoto M Metastasis-promoting activity of a novel molecule, Ag 243-5, derived from mycoplasma, and the complete nucleotide sequence Microbiol Immunol 1995 39 393 400 8551970 10.1111/j.1348-0421.1995.tb02218.x

[B20] Paton GR Jacobs JP Perkins FT Chromosome changes in human Diploid-cell cultures infected with mycoplasmas Nature 1965 207 43 45 10.1038/207043a0 5866523

[B21] Ketcham CM Anai S Reutzel R Sheng S Schuster SM Brenes RB Agbandje-McKenna M McKenna R Rosser CJ Boehlein SK p37 induces tumor invasiveness Mol Cancer Ther 2005 4 1031 1038 10.1158/1535-7163.MCT-05-0040 16020660

[B22] Steinemann C Fenner M Binz H Parish RW Invasive behavior of mouse sarcoma cells is inhibited by blocking a 37,000-dalton plasma membrane glycoprotein with Fab Fragments Proc Natl Acad Sci USA 1984 81 3747 3750 10.1073/pnas.81.12.3747 6587388 PMC345296

[B23] Ning JY Huang S Wu J Meng L Shou CC Protein p37 of *mycoplasma hyorhinis *induced secretion of TNF-α from human peripheral blood mononuclear cells Chinese Science Bulletin 2003 48 658 662 10.1360/03tb9141

[B24] Kita M Ohmoto Y Hirai Y Yamaguchi N Imanishi J Induction of cytokines in human peripheral blood mononuclear cells by mycoplasmas Microbiol Immunol 1992 36 507 516 1381037 10.1111/j.1348-0421.1992.tb02048.x

[B25] Kostyal DA Butler GH Beezhold DH *Mycoplasma hyorhinis *molecules that induce tumor necrosis factor α secretion by human monocytes Infect Immun 1995 63 3858 3863 7558292 10.1128/iai.63.10.3858-3863.1995 PMC173543

[B26] Dong ZW Yin WN Deng GR P40 antigen mediating inhibitory effect on the proliferation of ras transformed cells J Exp Clin Res 1994 13 331 337

[B27] Ilantzis C Thomson DM Michaelidou A Benchimol S Stanners CP Identification of a human cancer related organ-specific neoantigen Microbiol Immunol 1993 37 119 128 7684807 10.1111/j.1348-0421.1993.tb03188.x

[B28] Gilson E Alloing G Schmidt T Claverys JP Dudler R Hofnung M Evidence for a high affinity binding-protein dependent transport system in Gram-positive bacteria and in Mycoplasma EMBO J 1988 7 3971 3974 3208757 10.1002/j.1460-2075.1988.tb03284.x PMC454997

[B29] Steinemann C Fenner M Parish RW Binz H Studies of the invasiveness of the chemically induced mouse sarcoma FS9. I. Monoclonal antibodies to a 37,000 dalton membrane glycoprotein inhibit invasion of fibroblasts *in vitro* Int J Cancer 1984 34 407 414 10.1002/ijc.2910340319 6384068

[B30] Schmidhauser C Dudler R Schmidt T Parish RW A mycoplasmal protein influences tumour cell invasiveness and contact inhibition *in vitro* J Cell Sci 1990 95 499 506 2384527 10.1242/jcs.95.3.499

